# The safety and efficacy of neoadjuvant PD-1 inhibitor plus chemotherapy for patients with locally advanced gastric cancer: a systematic review and meta-analysis

**DOI:** 10.1097/JS9.0000000000002056

**Published:** 2024-08-22

**Authors:** Zhiyuan Yu, Chen Liang, Qixuan Xu, Zhen Yuan, Miao Chen, Rui Li, Sixin Zhou, Peiyu Li, Bo Wei, Xudong Zhao

**Affiliations:** a Medical School of Chinese PLA; b Department of General Surgery, The First Medical Center, Chinese PLA General Hospital, Beijing; c School of Medicine, Nankai University, Tianjin; d Department of Gastroenterology, Beijing Jishuitan Hospital, Capital Medical University, Beijing; e Department of Gastroenterology, The Second Affiliated Hospital of Shandong First Medical University, Shandong, People’s Republic of China

**Keywords:** locally advanced gastric cancer, meta-analysis, neoadjuvant chemotherapy, PD-1 inhibitor

## Abstract

**Background:**

The extensive utilization of immune checkpoint inhibitors (ICIs) targeting programmed cell death protein 1 (PD-1) has achieved significant advancements in the treatment of diverse solid tumors. The present meta-analysis aims to evaluate the safety and efficacy of neoadjuvant chemotherapy (NCT) plus PD-1 inhibitors for patients with locally advanced gastric cancer (LAGC).

**Methods:**

An electronic search of PubMed, EmBase, and the Cochrane Library was performed to identify the clinical trials of NCT + PD-1 inhibitor vs. NCT in patients with LAGC. The retrieval period extended from the establishment of the corresponding database until April 2024, and meta-analysis was conducted using Stata (version 15) software. Subsequently, direct comparative analysis was used to compare pooled results of neoadjuvant immunochemotherapy (NICT) with NCT.

**Results:**

After screening, six phase II/III randomized controlled trials (RCTs) and nine retrospective studies with 2953 patients were included. In meta-analysis, the NICT group demonstrated a significantly higher rate of pathological complete response (pCR) (*P*<0.001) and R0 resection (*P*=0.001), and a lower 2-year recurrence rate (*P*=0.001) compared to the NCT group. The NICT group, however, exhibited a higher incidence of severe treatment-related adverse events (TRAEs) (*P*=0.044). Additionally, the NICT and NCT groups exhibited no statistical differences in terms of the number of harvested lymph nodes, the occurrence of total TRAEs and postoperative complications, as well as the duration of postoperative hospitalization.

**Conclusions:**

The combination of PD-1 inhibitor + NCT in LAGC patients enhances the likelihood of achieving radical surgery and improves prognosis, albeit to some extent increasing the risk of severe TRAEs. NICT is anticipated to emerge as the preferred neoadjuvant therapy option for patients diagnosed with LAGC.

## Introduction

Highlights

- To our knowledge, this study represents the first comprehensive meta-analysis comparing the efficacy and safety of neoadjuvant immunochemotherapy (NICT) and neoadjuvant chemotherapy (NCT) in patients with locally advanced gastric cancer (LAGC).
- NICT demonstrated a significantly higher rate of pathological complete response (pCR) and R0 resection, and a lower 2-year recurrence rate.
- NICT exhibited comparable postoperative complications and hospital stay to NCT, while demonstrating a higher frequency of severe treatment-related adverse events (TRAEs).
- The combination of NCT and PD-1 inhibitors may be considered as a potential first-line neoadjuvant treatment option for LAGC.

The incidence and mortality rates of gastric cancer rank fifth and forth, respectively, among all malignant tumors worldwide, posing a significant threat to human life and health^1,2^. Due to the absence of specific early symptoms and signs, as well as a lack of awareness regarding early screening and diagnosis, a significant proportion of gastric cancer patients are diagnosed at an advanced stage, thereby missing the opportunity for curative surgery^3–5^. The utilization of neoadjuvant therapy, encompassing chemotherapy, radiotherapy and targeted therapy, has demonstrated efficacy in downstaging gastric cancer, augmenting the R0 resection rate, and enhancing patient prognosis^4,6,7^. The recent years have witnessed significant advancements in the treatment of various solid tumors through the widespread utilization of immune checkpoint inhibitors (ICIs), particularly programmed cell death protein 1 (PD-1)/programmed cell death-ligand 1 (PD-L1) inhibitors. The safety and efficacy of ICIs in neoadjuvant therapy, however, remains uncertain^7–9^.

Based on the results of the MAGIC trial, neoadjuvant chemotherapy (NCT) followed by D2 radical gastrectomy has been recognized as the standard treatment for locally advanced gastric cancer (LAGC)^10^. The subsequent application of capecitabine + oxaliplatin (XELOX), S-1 + oxaliplatin (SOX), and docetaxel + oxaliplatin + fluorouracil (FLOT) regimens in the neoadjuvant therapy of LAGC patients also demonstrated favorable efficacy^11,12^. The surgical pathological remission rate and long-term prognosis of LAGC patients remain unsatisfactory despite perioperative chemotherapy and surgical treatment, primarily due to tumor heterogeneity and other contributing factors. The results of the CheckMate-649 and ATTRACTION-04 trials demonstrated that the combination therapy of nivolumab (a PD-1 inhibitor) with chemotherapy exhibited superior efficacy compared to chemotherapy alone in patients with unresectable advanced gastric cancer^13,14^. However, there is a dearth of published literature on the application of PD-1 inhibitors in neoadjuvant therapy for LAGC, particularly regarding the comparative study between NCT + PD-1 inhibitor and NCT. Currently, there is a lack of meta-analysis comparing the efficacy of NCT + PD-1 inhibitor with NCT alone in the treatment of gastric cancer^15^.

## Materials and methods

### Data sources and search strategy

The present study has been reported in line with Preferred Reporting Items for Systematic Reviews and Meta-Analyses (PRISMA) (Supplemental Digital Content 1, http://links.lww.com/JS9/D347, Supplemental Digital Content 2, http://links.lww.com/JS9/D348)^16^ and assessing the methodological quality of systematic reviews (AMSTAR) (Supplemental Digital Content 3, http://links.lww.com/JS9/D349) Guidelines^17^. An electronic search of PubMed, EmBase, and the Cochrane Library was performed to identify the clinical trials of NCT + PD-1 inhibitors vs. NCT in patients with LAGC. The retrieval period extended from the establishment of the corresponding database until April 2024, and the language was limited to English. Medical subject headings (MeSH) and synonyms were combined for retrieval, and the detailed search strategy are exhibited in online Supplemental Material 1 (Supplemental Digital Content 4, http://links.lww.com/JS9/D350). The initial screening of the collected studies was performed by reviewing titles and abstracts. Subsequently, full texts were examined to identify the studies that satisfied the inclusion criteria. The inclusion of duplicate published trials was limited to the final or most comprehensive version, taking into account increasing patient numbers or extended follow-up durations.

### Study selection and data extraction

The eligibility for study inclusion in the meta-analysis was independently conducted by two authors. Besides, the study data were also independently extracted into standardized forms by two authors. If any disagreements arose, the team engaged in discussions and the third reviewers verified the consensus. Studies meeting the following criteria were included: (1) randomized controlled trials (RCTs), case–control studies, or cohort studies; (2) the enrolled patients were diagnosed with LAGC; (3) intervention and control groups treated with NCT + PD-1 inhibitors and NCT, respectively; (4) the physical state and tumor condition of the two groups were comparable. Exclusion criteria were as follows: (1) single-arm clinical trials, review articles, or case reports; (2) repeated reported studies; (3) gastric cancer without surgical intervention; (4) required outcome indicators were not reported.

The following data were extracted from included studies: author, publication year, study type, sample size, study period, cTNM tumor stage, PD-1 inhibitors and NCT regimes, pathological complete response (pCR), R0 resection, harvested lymph nodes, treatment-related adverse events (TRAEs), postoperative complications, postoperative hospital stay, and tumor recurrence. TRAEs referred to the adverse events induced by neoadjuvant therapy, which could be categorized into hematopoietic and nonhematopoietic systems based on their origin. The common TRAEs of the hematopoietic system were leukopenia, neutropenia, thrombocytopenia, and anemia, while those of the nonhematopoietic system were nausea, vomiting, and elevated transaminases. The postoperative complications referred to adverse events that occur as a result of surgical procedures, including but not limited to pulmonary or abdominal infections, gastrointestinal bleeding, gastrointestinal dysfunction, and anastomotic leakage. The occurrence of TRAEs and postoperative complications could be served as indicators for assessing the safety and impact of neoadjuvant therapy on surgery, respectively.

### Quality assessment

The Cochrane Collaboration’s tool and the Newcastle–Ottawa scale (NOS) were employed for evaluating the quality of RCTs and retrospective studies, respectively. The tool developed by the Cochrane Collaboration evaluate bias in six aspects, including selection, implementation, evaluation, follow-up, reporting, and other^18^. Subsequently, the quality of RCTs was categorized into high (6 points), fair (4–5 points), and low (0–3 points) based on the number of positive answers to the aforementioned six questions. In the NOS tool, three aspects were used to evaluate studies: selection (0–4 points), comparability (0–2 points), and exposure/outcome (0–3 points)^19^. The study was considered to be of satisfactory quality if it reaches or exceeds 6 points. All disagreements were resolved through discussions between the two commentators.

### Statistical analysis

All statistical analyses were conducted using Stata (version 15) software. A pairwise meta-analysis was implemented to compare the safety and efficacy between NCT + PD-1 inhibitors and NCT regimes. For dichotomous outcomes, pooled odds ratio (OR) and 95% CI were estimated using the Mantel–Haenszel method. Besides, for continuous outcomes, standardized mean difference (SMD) and 95% CI were estimated using the Cohen method. The method reported by Luo and Wan was used to convert the median and numerical range to mean and SD^20,21^. The *I* ^2^ and *Q* statistics were employed to evaluate the level of heterogeneity among the incorporated studies. *I* ^2^>30% or *P*<0.05 was considered substantial heterogeneity, random effects model was adopted; otherwise, a fixed effects model was used. Begg’s and Egger’s tests were performed to evaluate possible publication bias, and sensitivity analysis was performed to evaluate the robustness and reliability of the combined results.

## Results

### Eligible studies

**Figure 1:**
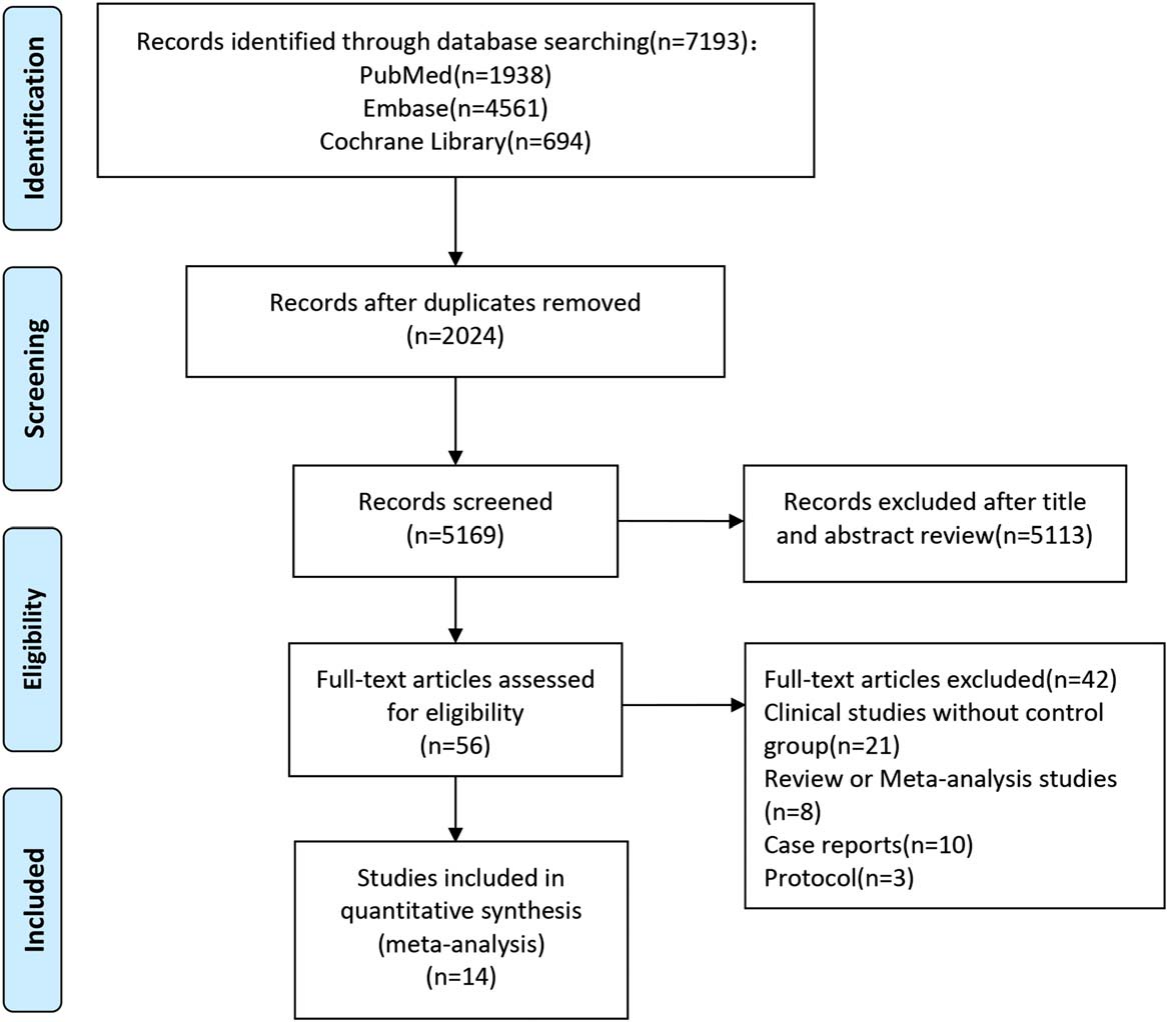
Flowchart of the studies screening and selection.

Based on the search strategy, a total of 7193 potential articles were identified. After screening, six phase II/III RCTs and nine retrospective studies (publication dates 2022–2024) with 2953 patients (1272 received NCT + PD-1 inhibitors and 1681 received NCT alone) were included in the final meta-analysis^22–35^ (Table 1). The flow diagram of literature screening is shown in Figure 1. The study conducted by Shitara *et al*.^22^ consisted of two distinct intervention cohorts (main cohort and FLOT cohort), therefore, we provided separate descriptions and analyses for each of these cohorts. In eligible studies, the NCT regimens included SOX, XELOX, FLOT, oxaliplatin+calcium levofolinate+ fluorouracil (FOLFOX), and other chemotherapy regimens. Patients in the NICT group received the aforementioned NCT regimens combined with intravenous PD-1 inhibitors, including pembrolizumab, camrelizumab, atezolizumab, nivolumab, toripalimab, sintilimab, or tislelizumab. The outcome indicators utilized in the meta-analysis are provided in online Supplemental Material 2 (Supplemental Digital Content 5, http://links.lww.com/JS9/D351) for each individual study.

**Table 1:** Summary of the 15 included clinical studies.

Study (Ref.) Year	Number of patients (NICT/NCT)	Study period	Tumor stage	PD-1 inhibitors regimes	NCT regimes	Quality score
Phase II/III randomized controlled trials (RCTs)						
Shitara^22^ 2024	402/402	2017–2021	cT_1-4_N_0-3_M_0_ (stage II–IVA)	Pembrolizumab, 200 mg. 21 days per cycle, three cycles	Cisplatin-based doublet chemotherapy (day 1: cisplatin, 80 mg/m^2^; days 1 to 14: capecitabine, 1000 mg/m², twice daily; or days 1 to 5: fluorouracil, 80 mg/m², per day). 21 days per cycle, 3 cycles	6
Shitara^22^ 2024	100/103	2017–2021		Pembrolizumab, 200 mg. 21 days per cycle, three cycles.	FLOT (docetaxel, 50 mg/m²; oxaliplatin, 85 mg/m²; fluorouracil, 2600 mg/m²; and leucovorin, 200 mg/m²). 14 days per cycle, four cycles	6
Lin^23^ 2024	51/53	2020–2022	cT_3-4_N_1-3_M_0_	Camrelizumab, 200 mg. 21 days per cycle, three cycles.	SAP (days 2 and 9: intravenous nab-paclitaxel 125 mg/m²; days 1 to 14: S-1, 40–60 mg depending on body surface area, twice daily). Apatinib, oral, 250 mg/d. 21 days per cycle, three cycles	4
Peng^24^ 2024	21/21	Unknown -2023		Atezolizumab, 1200 mg. 21 days per cycle, three cycles.	XELOX (day 1: oxaliplatin, 130 mg/m^2^; days 1 to 14: capecitabine, 1000 mg/m^2^, twice daily). Trastuzumab, 6 mg/kg. 21 days per cycle, three cycles	4
Lorenzen^25^ 2023	146/149	2018–2020	cT_1-4_N_0-3_M_0_	Atezolizumab, 840 mg. 14 days per cycle, four cycles	FLOT (day 1: docetaxel 50 mg/m²; oxaliplatin 85 mg/m²; leucovorin 200 mg/m²; and fluorouracil 2600 mg/m²). 14 days per cycle, four cycles	4
Min^26^ 2022	33/30	2020–2022		Camrelizumab, 200 mg. 21 days per cycle, four cycles.	FLOT. Four cycles.	4
Retrospective clinical studies (non-RCTs)						
Bao^27^ 2024	28/61	2020–2021	cT_1-4_N_0-3_M_0-1_ (stage I–IV)	PD-1 mAb, 200 mg.	SOX (day 1: oxaliplatin, 130 mg/m^2^; days 1 to 14: S-1, surface area ≥1.5 m^2^, 120 mg/d, <1.25 m^2^, 80 mg/d, surface area 1.25–1.5 m², 100 mg/d, twice daily). 21 days per cycle.	6
Cui^28^ 2024	49/86	2020–2023	cT_2_N_1-3_M_0_/cT_3-4b_N_0-3_M_0_ (stage II–IVA)	Nivolumab, pembrolizumab, toripalimab, camrelizumab, or sintilimab	Dual-drug NCT regimen, such as SOX, XELOX, and SAP, or triple-drug NCT regimens, such as FLOT and FOLFOX. 2–8 cycles	7
Lin^29^ 2024	38/90	2019–2020	cT_4_N_0-3_M_0_ (stage IIB–IVA)	Camrelizumab, 200 mg.	SAP (day 1: intravenous nab-paclitaxel 260 mg/m² over 30 min; days 1 to 14: S-1, surface area ≥1.5 m^2^, 120 mg/d, <1.25 m^2^, 80 mg/d, surface area 1.25–1.5 m², 100 mg/d, twice daily) 21 days per cycle	8
Sun^30^ 2024	195/390	2016–2022	cT_3-4b_N_0-3_M_0_ (stage II–IVA)	Sintilimab, nivolumab, or camrelizumab	Dual-drug NCT regimen, such as SOX, XELOX, SAP, and DS, or triple-drug NCT regimens, such as FLOT, DOS, and POF	8
Jiang^31^ 2023	50/119	2012–2021	cT_2-4_N_0-3_M_0_ (stage II–III)	Tislelizumab, 200 mg. 21 days per cycle	SOX (day 1: oxaliplatin, 130 mg/m^2^; days 1 to 14: S-1, 40 mg/m^2^, twice daily), 21 days per cycle. or FOLFOX (day 1: oxaliplatin, 85 mg/m^2^; leucovorin, 400 mg/m^2^; fluorouracil, 400 mg/m^2^, followed by 2400 mg/m^2^ in 46 h). 14 days per cycle	7
Su^32^ 2023	30/50	2019–2021	cT_1-4b_N_0-3_M_0-1_ (stage II-IVB)	Pembrolizumab, 200 mg; toripalimab, 3 mg/kg; carrelizumab, 200 mg; or sintilimab, 200 mg. 21 days per cycle	XELOX (day 1: oxaliplatin, 130 mg/m^2^; days 1 to 14: capecitabine, 1000 mg/m^2^, twice daily), 21 days per cycle. or FOLFOX (day 1: oxaliplatin, 85 mg/m^2^; leucovorin, 400 mg/m^2^; fluorouracil, 400 mg/m^2^, followed by 2400 mg/m^2^ in 46 h), every 2 weeksk. or FLOT (day 1: docetaxel, 50 mg/m^2^; oxaliplatin, 85 mg/m^2^; leucovorin, 200 mg/m^2^; fluorouracil, 2600 mg/m^2^, 24 h continuous infusion), 21 days per cycle. 2–8 cycles	6
Wang^33^ 2023	39/34	2019–2022	cT_3-4a_N_1-3_M_0_ (stage III)	Sintilimab, 200 mg; carrelizumab, 200 mg; or toripalimab, 240 mg. 21 days per cycle, three cycles	SOX, or XELOX. Apatinib, oral, 375 mg/d. 21 d per cycle, three cycles	7
Xiong^34^ 2023	56/50	2019–2023	cT_3-4a_N_1-3_M_0_ (stage III)	Sintilimab, 200 mg; or camrelizumab, 200 mg. 21 days per cycle, four cycles	SOX, or XELOX. Apatinib, oral, 250 mg/d. 21 days per cycle, four cycles	6
Zhang^35^ 2023	34/43	2019–2021	cT_3-4a_N_0-3_M_0_ (stage II–III)	Camrelizumab, 200 mg, sintilimab, 200 mg, toripalimab, 240 mg, 21 days per cycle; or nivolumab, 3 mg/kg, 14 days per cycle. 2–4 cycles	SOX, or FLOT. 2–4 cycles	7

DOS, docetaxel+oxaliplatin+S-1; DS, docetaxel+S-1; FLOT, fluorouracil+leucovorin+oxaliplatin+docetaxel; FOLFOX, oxaliplatin+calcium levofolinate+fluorouracil; NCT, neoadjuvant chemotherapy; NICT, neoadjuvant immunotherapy combined with chemotherapy; PD-1, programmed cell death 1; POF, paclitaxel+oxaliplatin+fluorouracil; RCTs, randomized controlled trials; SAP, S-1+nab-paclitaxel; SOX, S-1+oxaliplatin; XELOX, capecitabine+oxaliplatin.

### Meta-analysis results

#### pCR and R0 resection

**Figure 2:**
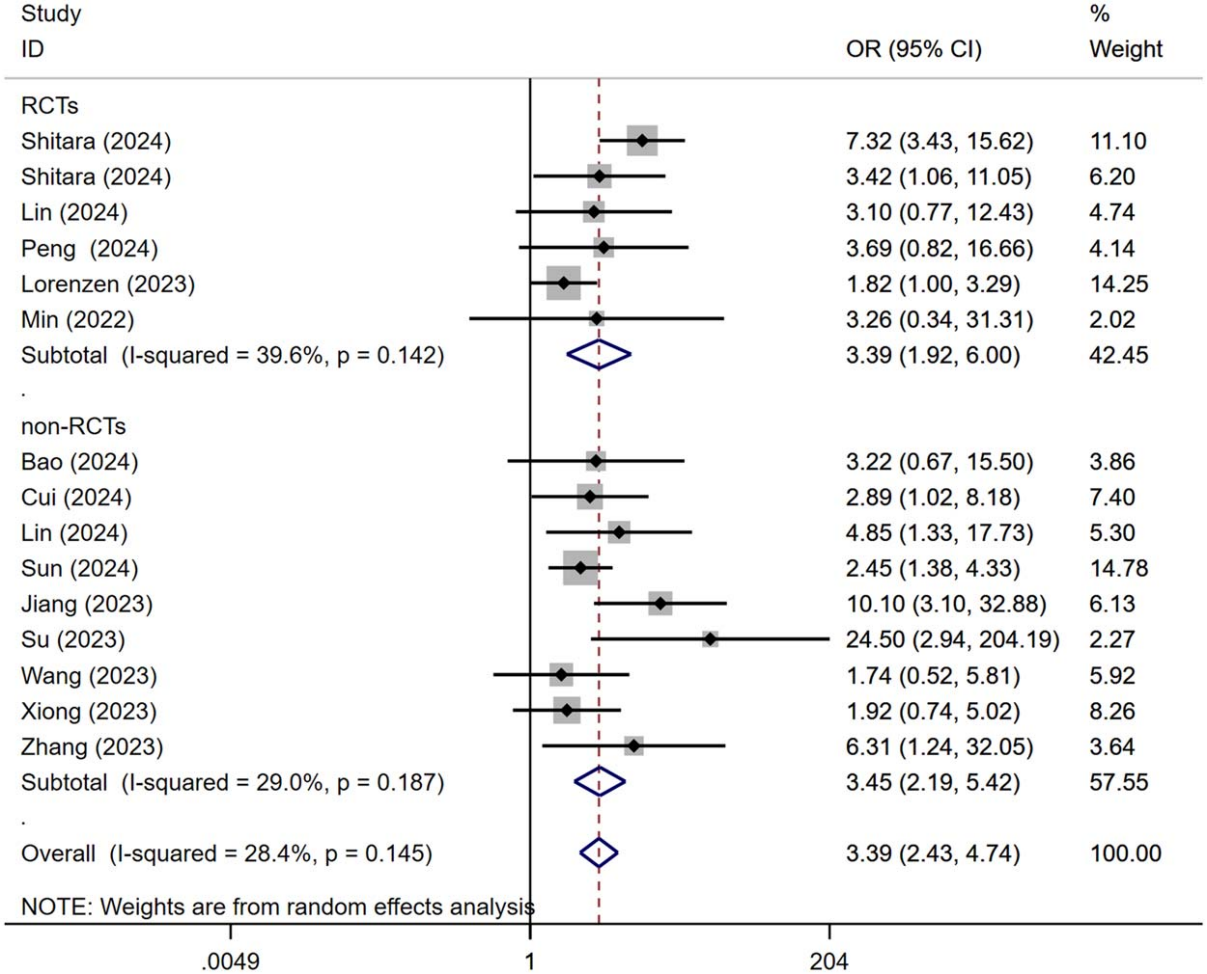
Forest plot showing the odds ratio in pCR rate. pCR, pathological complete response.

The rate of pCR was reported in all included studies. Fifteen included studies were categorized into RCTs and non-RCTs subgroups for pairwise meta-analysis, based on the study type. The utilization of a random effects model was warranted due to the observed heterogeneity among RCTs (*I* ^2^=39.6%, *P*=0.142). Meta-analysis results revealed that patients in the NICT group exhibited a significantly higher rate of achieving pCR (OR=3.393, 95% CI: 2.428–4.740, *P*<0.001) (Fig. 2).

**Figure 3:**
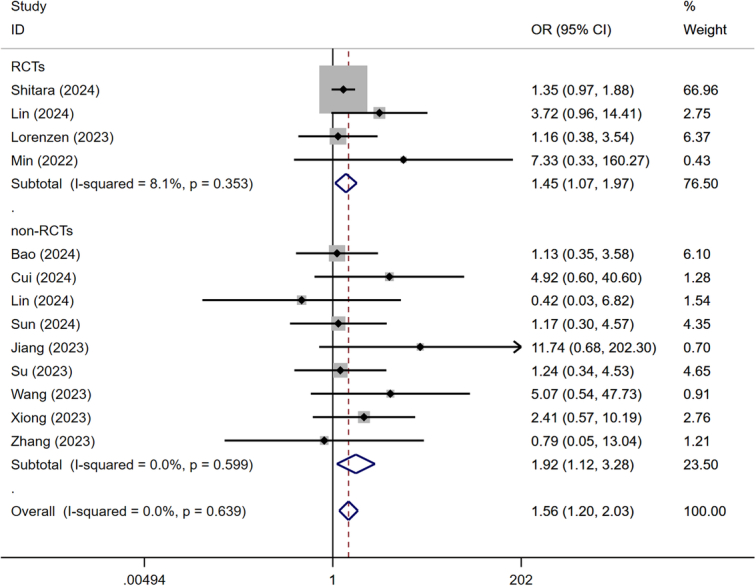
Forest plot showing the odds ratio in R0 resection rate.

Four RCTs and nine non-RCTs reported the rate of R0 resection. No significant heterogeneity was observed among the included studies, thus the fixed-effect model was utilized. The results of pairwise meta-analysis revealed a significantly higher rate of R0 resection in the NICT group compared to the NCT group (OR=1.560, 95% CI: 1.198-2.032, *P*=0.001) (Fig. 3).

#### TRAEs

**Figure 4:**
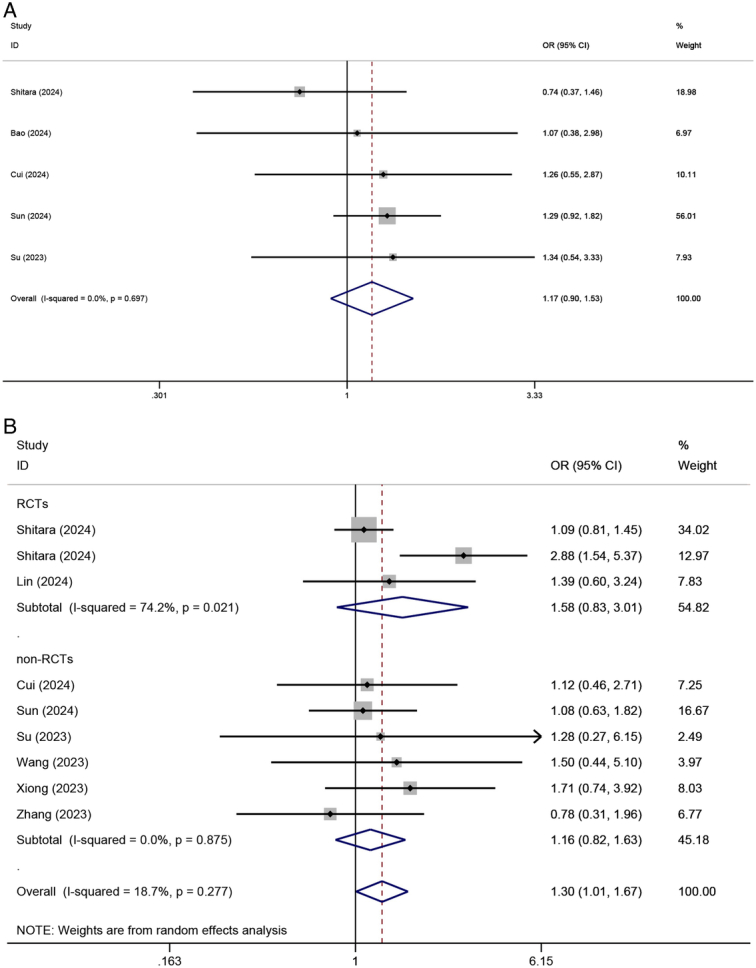
Forest plot showing the odds ratio in (A) total and (B) severe TRAEs between NICT and NCT. TRAEs, treatment-related adverse events; NICT, neoadjuvant immunochemotherapy; NCT, neoadjuvant chemotherapy.

Five trials totaling 1693 patients reported the total TRAEs. No heterogeneity was seen among the included studies (*I* ^2^=0%, *P*=0.697). The fixed-effect meta-analysis showed no significant difference in the total TRAEs rate between NICT and NCT groups (OR=1.173, 95% CI: 0.900–1.528, *P*=0.238) (Fig. 4A). Furthermore, the rate of severe (Grade III–V) TRAEs was reported in nine studies (three RCTs and six non-RCTs), with 956 patients in the NICT group and 1211 patients in the NCT group, respectively. The neoadjuvant regimens utilized in the three RCTs, specifically pembrolizumab+cisplatin-based doublet chemotherapy, pembrolizumab+FLOT, and camrelizumab+SAP, exhibited respective incidences of severe TRAEs at 64.4% (259/402), 42.0% (42/100), and 33.3% (17/51). Statistical heterogeneity was observed in the RCTs subgroup (*I* ^2^=74.2%, *P*=0.021). The comparative meta-analysis conducted using random-effect revealed a higher rate of severe TRAEs in the NICT group (OR=1.297, 95% CI: 1.007–1.670, *P*=0.044) (Fig. 4B).

#### Harvested lymph nodes

**Figure 5:**
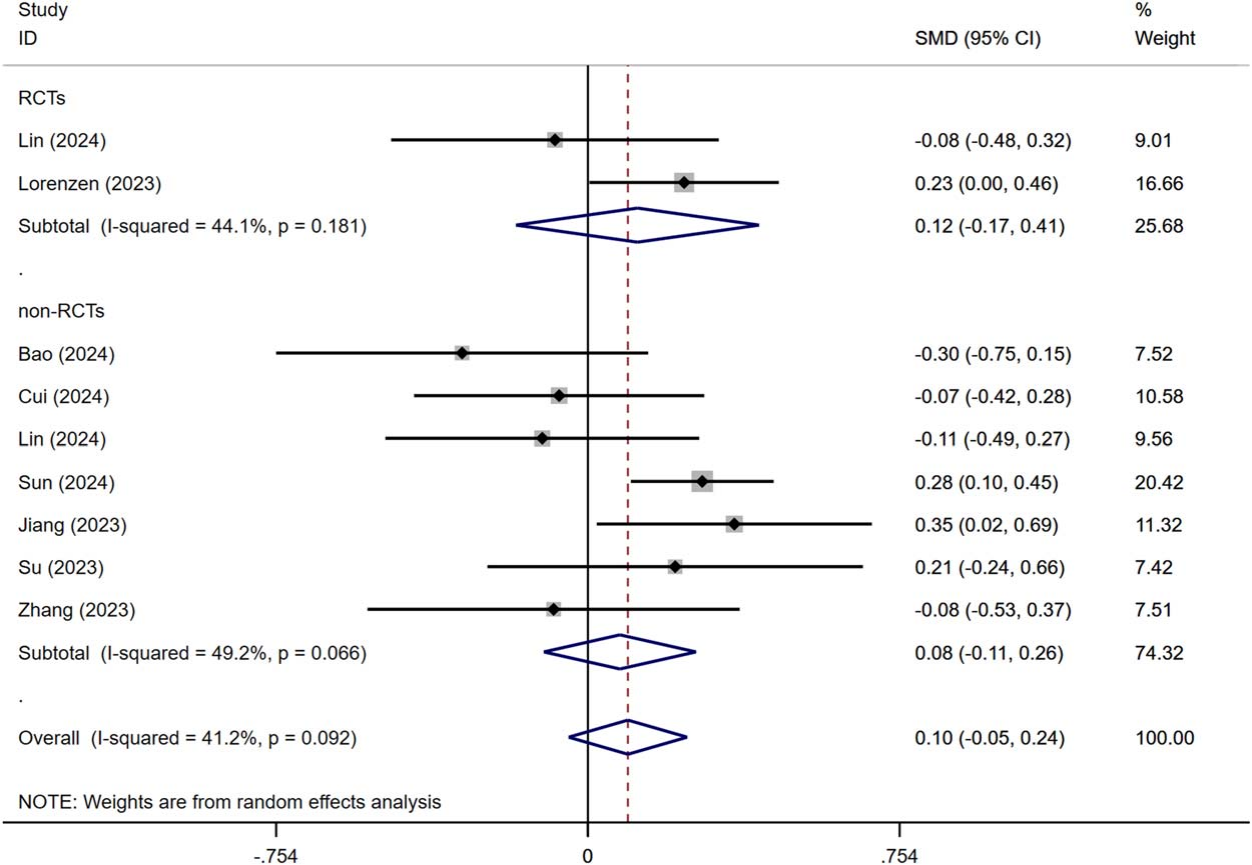
Forest plot showing the standardized mean difference in harvested lymph nodes between NICT and NCT. NICT, neoadjuvant immunochemotherapy; NCT, neoadjuvant chemotherapy.

Nine trials containing 1662 patients reported the number of harvested lymph nodes. Significant heterogeneity was observed among the included studies (*I* ^2^=41.2%, *P*=0.092), thus the random-effect model was adopted. The comparative meta-analysis showed no statistical differences (SMD=0.097, 95% CI: −0.046–0.240, *P*=0.185) (Fig. 5).

#### Postoperative complications

**Figure 6:**
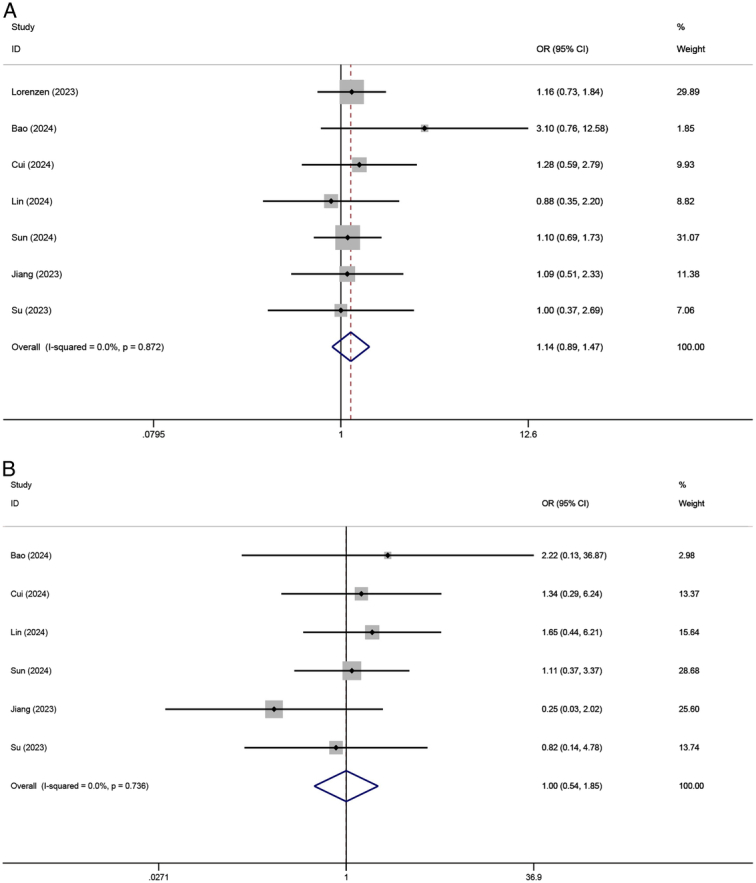
Forest plot showing the odds ratio in (A) total and (B) severe postoperative complications between NICT and NCT. NICT, neoadjuvant immunochemotherapy; NCT, neoadjuvant chemotherapy.

The meta-analysis of total (*I* ^2^=0%, *P*=0.872) and severe (Grade III–V) postoperative complications (*I* ^2^=0%, *P*=0.736) both showed no significant heterogeneity among the included studies. Thus, the fixed-effect model was employed. The comparative meta-analysis for total (OR=1.143, 95% CI: 0.888–1.472, *P*=0.299) (Fig. 6A) and severe complications (OR=0.999, 95% CI: 0.540–1.846, *P*=0.997) (Fig. 6B) both showed no statistical differences.

#### Postoperative hospital stay and 2-year recurrence

**Figure 7:**
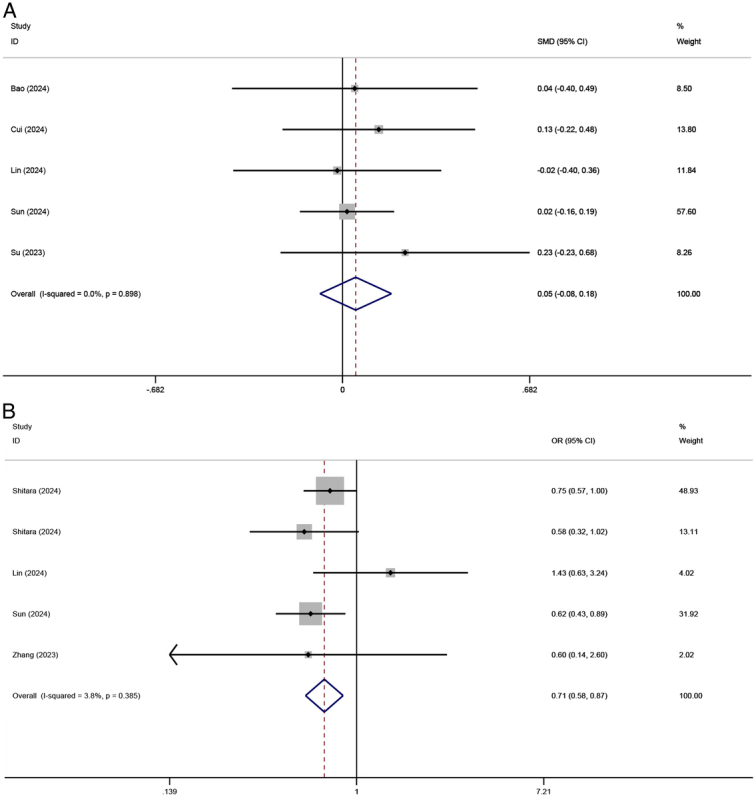
Forest plot for (A) postoperative hospital stay and (B) 2-year recurrence between NICT and NCT. NICT, neoadjuvant immunochemotherapy; NCT, neoadjuvant chemotherapy.

Five RCTs totaling 1017 patients reported the postoperative hospital stay, and no heterogeneity was seen among the included studies (*I* ^2^=0%, *P*=0.898). The fixed-effect meta-analysis showed no significant difference between NICT and NCT groups (SMD=0.048, 95% CI: −0.082 to 0.178, *P*=0.471) (Fig. 7A). Additionally, the 2-year recurrence rate was reported in 5 studies, with 749 patients in the NICT and 1005 patients in the NCT groups, respectively. Among these studies, the efficacy of specific PD-1 inhibitors and chemotherapy regimens was evaluated in two RCTs and one non-RCT studies. As reported in these studies, the 2-year recurrence rates for patients receiving pembrolizumab+cisplatin-based doublet chemotherapy, pembrolizumab+FLOT, and camrelizumab+SAP regimens were 42.0% (169/402), 32.0% (32/100), and 34.2% (13/38), respectively. There was no statistical heterogeneity between studies (I^2^=3.8%, *P*=0.385). The meta-analysis based on the fixed-effect model showed that the patients in the NICT group experiencing a lower 2-year recurrence rate compared to those in the NCT group (OR=0.711, 95% CI: 0.583–0.867, *P*=0.001) (Fig. 7B).

### Publication bias and sensitivity analysis

**Figure 8:**
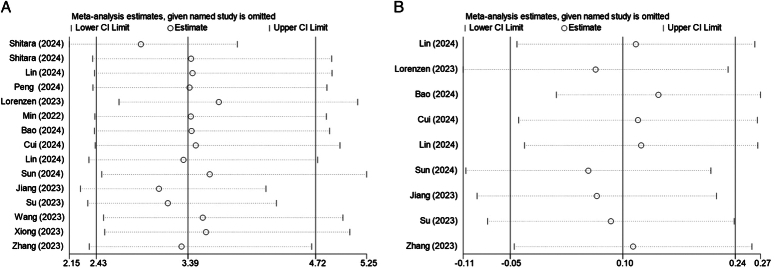
Sensitivity analysis of (A) pCR rate and (B) harvested lymph nodes. pCR, pathological complete response.

The results of Begg and Egger tests both showed no evidence of publication bias for pCR, R0 resection, total and severe TRAEs, total and severe postoperative complications, 2-year recurrence, or postoperative hospital stay. Although the Begg test for harvested lymph nodes showed no evidence of publication bias (*P*=0.251), the Egger test showed potential evidence of publication bias (*P*=0.025) (Table 2). The Begg’s funnel plots for pCR and R0 resection were also drawn (Supplemental Material 3A-B, Supplemental Digital Content 6, http://links.lww.com/JS9/D352). Sensitivity analysis was performed for pCR and harvested lymph nodes, and no relative significant difference was noticed after systematically eliminating each study, demonstrating the stability of our findings (Fig. 8).

**Table 2:** Begg and Egger test for publication bias.

Outcomes	*P* for Begg	*P* for Egger
pCR	0.113	0.095
R0 resection	0.127	0.122
Total TRAEs	0.806	0.550
Severe TRAEs	0.917	0.487
Harvested lymph nodes	0.251	0.025
Total postoperative complications	0.764	0.384
Severe postoperative complications	0.707	0.615
Postoperative hospital stay	0.221	0.316
2-year recurrence	0.806	0.788

pCR, pathological complete response; TRAEs, treatment-related adverse events.

## Discussion

The previous studies have demonstrated that tumor cells possess various mechanisms to evade the host immune response, with the most prevalent being the regulation of immune checkpoints to suppress T-cell activity and proliferation. This leads to the establishment of an immunosuppressive microenvironment, thereby preventing recognition and attack by the immune system^36^. The ICIs therapy primarily employs monoclonal antibodies to specifically bind to the immune checkpoints present on the surface of tumor or immune cells, thereby obstructing negative regulation and reactivating the immune system for effective eradication of tumor cells. As a breakthrough in cancer treatment, immunotherapy has emerged as an additional efficacious modality following surgery, chemoradiotherapy, and targeted therapy. Besides, it has gained widespread utilization across various malignancies including lung and gastric cancers^37,38^.

PD-1, which is expressed on the surface of T, B, and myeloid cells, functions as an inhibitory regulator in the immune system. The immune system’s capacity to surveil, identify, and eliminate tumor cells can be restored by targeting this molecule with inhibitors. The effectiveness of monotherapy with anti-PD-1 antibodies has been substantiated by numerous studies in the treatment of advanced gastric cancer^39,40^. Based on the positive findings of the KEYNOTE-059 study cohort, the FDA has granted approval for the use of pabolizumab in patients with PD-L1-positive gastric cancer and gastroesophageal junction cancer (GEJC) who have not responded to previous treatment^39^. The results of the ATTRACTION-04 study also demonstrated that chemotherapy plus nivolumab significantly extended disease-free survival (DFS) and increased the objective remission rate in patients with advanced metastatic gastric cancer, compared to chemotherapy alone^13^. At present, the combination of chemotherapy and immunotherapy (ICIs) has received approval as a first-line treatment option for patients with unresectable, metastatic, or advanced gastric cancer.

Based on previous literature reports regarding the precise efficacy of ICIs in the first-line treatment of gastric cancer, numerous domestic and international institutions have sequentially conducted diverse combination therapies involving ICIs for neoadjuvant therapy in gastric cancer. The KEYNOTE-585 study was a multicenter, randomised, placebo-controlled, double-blind, phase Ⅲ study conducted at 143 medical centers in 24 countries. Its objective was to assess the effect of neoadjuvant and adjuvant pembrolizumab plus chemotherapy in LAGC or GEJC. An interim analysis of this study revealed that neoadjuvant and adjuvant pembrolizumab demonstrated a significant improvement in pCR compared to placebo; however, it did not yield a statistically significant enhancement in patients’ event-free survival^22^. Additionally, a phase II, single-center, two-arm study (ChiCTR2000030610) recruited 61 patients diagnosed with cT3-4aN+M0 gastric cancer or GEJC and randomly allocated them into the neoadjuvant FLOT or neoadjuvant camrelizumab+FLOT groups. The findings of this study suggested that the combination of immunotherapy and chemotherapy may lead to superior outcomes in terms of achieving R0 resection, pCR, and postoperative node-negative rate compared to chemotherapy alone; however, no statistically significant differences were reported between the two groups^26^.

In summary, preliminary findings from several phase I–III clinical trials for LAGC/GEJC have demonstrated promising outcomes when neoadjuvant treatment regimens of PD-1 inhibitors are combined with chemotherapy. The majority of the published literature, however, consists of single-arm clinical trials and is deficient in control groups^8,9,15,41^. Additionally, due to the delayed initiation and prolonged patient recruitment period, the available reports from large-scale RCTs primarily consist of preliminary trial data, thereby limiting their clinical utility^42,43^. After conducting a thorough search of the database, we discovered only one meta-analysis on NICT. However, it is worth noting that this meta-analysis included a limited sample size of just 206 patients and lacked comprehensive outcome indicators. Furthermore, there was a notable absence of comparative analysis with NCT^15^. In the period of 2023–2024, numerous high-quality phase II/III RCTs internationally have reported the preliminary and interim results comparing NCT + PD-1 inhibitors with NCT^22–26^. Besides, a substantial number of retrospective clinical studies were also emerged^27–35^. Consequently, the execution of our meta-analysis holds immense importance in augmenting clinical evidence and providing guidance for clinical practice.

This meta-analysis comprised 15 trials encompassing a total of 2953 patients, with 12 of these trials specifically conducted within Chinese gastric cancer populations. The incidence and mortality rates of gastric cancer in China rank third among all malignant tumors, with the highest number of new cases reported annually worldwide. Additionally, the detection rate of early gastric cancer (EGC) in China is relatively low, accounting for less than 20% of all cases, with the majority being diagnosed at advanced stages. Due to the substantial population base of LAGC patients, the implementation and promotion of neoadjuvant therapy programs in China can be initiated at an early stage, providing favorable conditions for the development of clinical trials. Our findings demonstrated that the utilization of PD-1 inhibitors + NCT was associated with increased rates of pCR, R0 resection, and 2-year DFS. The action mechanism of chemotherapy drugs involves targeting DNA, RNA, proteins, enzymes, and other cellular components to induce tumor cell death. The antitumor efficacy of ICIs is achieved by impeding the immune evasion process and reinstating the body’s immune response. The immune pattern may be modulated by the mechanism of antitumor action exhibited by traditional chemotherapy drugs. When ICIs are combined with chemotherapy, on the one hand, chemotherapy drugs can enhance the antitumor immune response of patients; on the other hand, ICIs can augment chemotherapy sensitivity by boosting the antitumor immune response and further eliminate tumor cells that develop resistance after chemotherapy^44,45^. To summarize, the synergistic effect of above two distinct antitumor mechanisms can enhance the cytotoxicity towards tumor cells, augment the likelihood of achieving pCR, diminish tumor staging, increase the probability of radical surgery, and ultimately ameliorate prognosis. Furthermore, with the continuous emergence of anti-HER-2 and VEGFR drugs, along with the announcement of several research results on ICIs combined with targeted therapy, there is an expectation for significant advancements in neoadjuvant therapy for patients with HER-2 and VEGFR-positive gastric cancer^46,47^.

The efficacy of drug therapy for cancer should not be the sole focus; attention must also be given to its safety, encompassing the occurrence of TRAEs and complications. The chemotherapeutic drugs utilized in clinical settings are cytotoxic agents, which induce varying degrees of damage to normal cells and consequently manifest diverse toxicities and side effects. Myelosuppression, hepatorenal dysfunction, gastrointestinal disturbances, and hand-foot syndrome are the common adverse reactions following chemotherapy. The administration of immunotherapy, while effectively inducing durable tumor remission in patients with malignant tumors, is also associated with the occurrence of adverse events. These events are primarily attributed to the up-regulation of inflammatory responses triggered by immune cell release through immune checkpoints^48,49^. In this study, the total TRAEs observed in the NICT group were comparable to those in the NCT group; however, a higher incidence of severe TRAEs was noted in the NICT group. Therefore, it is imperative to closely monitor changes in vital signs during the application of the NICT regime, remain vigilant against severe TRAEs, and promptly formulate and adjust treatment plans based on patients’ conditions. Besides, the effect of NICT on severe TRAEs requires further investigation, as subgroup analyses did not reveal statistical differences. It was also found that the combination of NCT and PD-1 inhibitors had no impact on surgical complexity, postoperative recovery time, or complications, suggesting the safety of immunochemotherapy.

The present study, despite its systematic collection of published clinical studies and comprehensive analysis of numerous clinical indicators, still has several inherent limitations. Firstly, the majority of included studies were conducted exclusively on the Chinese population, thus necessitating further investigation into the generalizability of this analysis. In addition, the outcome data of the RCTs group represented the preliminary and interim findings from phase II/III clinical trials, featuring incomplete outcome indicators and limited follow-up duration. The comprehensive outcomes and long-term prognostic data necessitate supplementation through subsequent studies. Last but not the least, the types, dosages, and frequencies of chemotherapy drugs or PD-1 inhibitors varied across each RCT study, while diverse combinations of chemotherapy or PD-1 inhibitor drugs were employed in each non-RCT study. Therefore, conducting subgroup analyses based on specific medication regimens to identify optimal combinations of chemotherapy regimens and PD-1 inhibitors with enhanced efficacy and safety is not feasible. It is anticipated that the findings from additional phase III and large-scale multicenter RCTs can complement and validate the results obtained from this analysis.

## Conclusions

The patients treated with a combination of NCT and PD-1 inhibitors demonstrated a significantly higher rate of achieving pCR and R0 resection, while experiencing a lower 2-year recurrence rate compared to those who received NCT alone. The NICT group, however, exhibited a higher incidence of severe TRAEs. Additionally, there were no statistically significant differences observed in terms of the number of harvested lymph nodes, the occurrence of postoperative complications, as well as the duration of postoperative hospitalization. The neoadjuvant treatment regimen of combining PD-1 inhibitor with chemotherapy has demonstrated promising clinical efficacy in LAGC, albeit with an increased incidence of severe TRAEs. Thus, the implementation of NICT requires the proactive development of prevention and treatment strategies to address potential severe TRAEs.

## Ethical approval

This is a meta-analysis of published data, thus no ethical approval was required.

## Consent

Not applicable.

## Source of funding

The authors declare no source of funding.

## Author contribution

X.Z., B.W., and P.L.: contributed to protocol/project development and manuscript writing/editing; Z.Y., C.L., Q.X., and Z.Y.: contributed to statistical analyses, results interpretation, and manuscript writing/editing; M.C., R.L., and S.Z.: contributed to literature search and data collection. All authors have read and approved the final manuscript.

## Conflicts of interest disclosure

The authors declare that they have no conflicts of interest to disclosure.

## Research registration unique identifying number (UIN)

1. Name of the registry: PROSPERO database.

2. Unique Identifying number or registration ID: CRD42024550431.

3. Hyperlink to your specific registration (must be publicly accessible and will be checked):https://www.crd.york.ac.uk/prospero/display_record.php?ID=CRD42024550431.

## Guarantor

Xudong Zhao, Peiyu Li, and Bo Wei.

## Data availability statement

All data generated or analyzed and software used during this study are included in the article/Supplementary Material. Further inquiries can be directed to the corresponding author.

## Provenance and peer review

Not commissioned, externally peer-reviewed.

## Supplementary Material

## References

[1] Sung H Ferlay J Siegel RL. Global Cancer Statistics 2020: GLOBOCAN estimates of incidence and mortality worldwide for 36 cancers in 185 countries. CA Cancer J Clin 2021;71:209–249. 33538338 10.3322/caac.21660

[2] Guan WL He Y Xu RH. Gastric cancer treatment: recent progress and future perspectives. J Hematol Oncol 2023;16:57. 37245017 10.1186/s13045-023-01451-3 PMC10225110

[3] Wang FH Zhang XT Li YF. The Chinese Society of Clinical Oncology (CSCO): Clinical guidelines for the diagnosis and treatment of gastric cancer, 2021. Cancer Commun (Lond) 2021;41:747–795. 34197702 10.1002/cac2.12193 PMC8360643

[4] Ajani JA D’Amico TA Bentrem DJ. Gastric Cancer, Version 2.2022, NCCN clinical practice guidelines in oncology. J Natl Compr Canc Netw 2022;20:167–192. 35130500 10.6004/jnccn.2022.0008

[5] Li GZ Doherty GM Wang J. Surgical management of gastric cancer: a review. JAMA Surg 2022;157:446–454. 35319717 10.1001/jamasurg.2022.0182

[6] Trumbull DA Lemini R Diaz Vico T. Prognostic significance of complete pathologic response obtained with chemotherapy versus chemoradiotherapy in gastric cancer. Ann Surg Oncol 2021;28:766–773. 32737698 10.1245/s10434-020-08921-9

[7] Joshi SS Badgwell BD. Current treatment and recent progress in gastric cancer. CA Cancer J Clin 2021;71:264–279. 33592120 10.3322/caac.21657 PMC9927927

[8] Li S Xu Q Dai X. Neoadjuvant therapy with immune checkpoint inhibitors in gastric cancer: a systematic review and meta-analysis. Ann Surg Oncol 2023;30:3594–3602. 36795255 10.1245/s10434-023-13143-w

[9] Hasegawa H Shitara K Takiguchi S. A multicenter, open-label, single-arm phase I trial of neoadjuvant nivolumab monotherapy for resectable gastric cancer. Gastric Cancer 2022;25:619–628. 35254550 10.1007/s10120-022-01286-w PMC9013329

[10] Cunningham D Allum WH Stenning SP. Perioperative chemotherapy versus surgery alone for resectable gastroesophageal cancer. N Engl J Med 2006;355:11–20. 16822992 10.1056/NEJMoa055531

[11] Yu J Gao Y Chen L. Effect of S-1 plus oxaliplatin compared with fluorouracil, leucovorin plus oxaliplatin as perioperative chemotherapy for locally advanced, resectable gastric cancer: a randomized clinical trial. JAMA Netw Open 2022;5:e220426. 35226081 10.1001/jamanetworkopen.2022.0426 PMC8886520

[12] Al-Batran SE Homann N Pauligk C. Perioperative chemotherapy with fluorouracil plus leucovorin, oxaliplatin, and docetaxel versus fluorouracil or capecitabine plus cisplatin and epirubicin for locally advanced, resectable gastric or gastro-oesophageal junction adenocarcinoma (FLOT4): a randomised, phase 2/3 trial. Lancet 2019;393:1948–1957. 30982686 10.1016/S0140-6736(18)32557-1

[13] Kang YK Chen LT Ryu MH. Nivolumab plus chemotherapy versus placebo plus chemotherapy in patients with HER2-negative, untreated, unresectable advanced or recurrent gastric or gastro-oesophageal junction cancer (ATTRACTION-4): a randomised, multicentre, double-blind, placebo-controlled, phase 3 trial. Lancet Oncol 2022;23:234–247. 35030335 10.1016/S1470-2045(21)00692-6

[14] Janjigian YY Shitara K Moehler M. First-line nivolumab plus chemotherapy versus chemotherapy alone for advanced gastric, gastro-oesophageal junction, and oesophageal adenocarcinoma (CheckMate 649): a randomised, open-label, phase 3 trial. Lancet 2021;398:27–40. 34102137 10.1016/S0140-6736(21)00797-2 PMC8436782

[15] Xu H Li T Shao G. Evaluation of neoadjuvant immunotherapy plus chemotherapy in Chinese surgically resectable gastric cancer: a pilot study by meta-analysis. Front Immunol 2023;14:1193614. 37426646 10.3389/fimmu.2023.1193614 PMC10326549

[16] Page MJ McKenzie JE Bossuyt PM. The PRISMA 2020 statement: an updated guideline for reporting systematic reviews. BMJ 2021;372:n71. 33782057 10.1136/bmj.n71 PMC8005924

[17] Shea BJ Reeves BC Wells G. AMSTAR 2: a critical appraisal tool for systematic reviews that include randomised or non-randomised studies of healthcare interventions, or both. BMJ 2017;358:j4008. 28935701 10.1136/bmj.j4008 PMC5833365

[18] Higgins JP Altman DG Gøtzsche PC. The Cochrane Collaboration’s tool for assessing risk of bias in randomised trials. BMJ 2011;343:d5928. 22008217 10.1136/bmj.d5928 PMC3196245

[19] Stang A. Critical evaluation of the Newcastle-Ottawa scale for the assessment of the quality of nonrandomized studies in meta-analyses. Eur J Epidemiol 2010;25:603–605. 20652370 10.1007/s10654-010-9491-z

[20] Luo D Wan X Liu J. Optimally estimating the sample mean from the sample size, median, mid-range, and/or mid-quartile range. Stat Methods Med Res 2018;27:1785–1805. 27683581 10.1177/0962280216669183

[21] Wan X Wang W Liu J. Estimating the sample mean and standard deviation from the sample size, median, range and/or interquartile range. BMC Med Res Methodol 2014;14:135. 25524443 10.1186/1471-2288-14-135 PMC4383202

[22] Shitara K Rha SY Wyrwicz LS. KEYNOTE-585 investigators. Neoadjuvant and adjuvant pembrolizumab plus chemotherapy in locally advanced gastric or gastro-oesophageal cancer (KEYNOTE-585): an interim analysis of the multicentre, double-blind, randomised phase 3 study. Lancet Oncol 2024;25:212–224. 38134948 10.1016/S1470-2045(23)00541-7

[23] Lin JX Tang YH Zheng HL. Neoadjuvant camrelizumab and apatinib combined with chemotherapy versus chemotherapy alone for locally advanced gastric cancer: a multicenter randomized phase 2 trial. Nat Commun 2024;15:41. 38167806 10.1038/s41467-023-44309-5 PMC10762218

[24] Peng Z Zhang X Liang H. Atezolizumab and trastuzumab plus chemotherapy in patients with HER2+ locally advanced resectable gastric cancer or adenocarcinoma of the gastroesophageal junction: A multicenter, randomized, open-label phase II study. J Clin Oncol 2024;42:312.37931206

[25] Lorenzen S Götze TO Thuss-Patience P. Perioperative atezolizumab plus fluorouracil, leucovorin, oxaliplatin, and docetaxel for resectable esophagogastric cancer: interim results from the randomized, multicenter, phase II/III DANTE/IKF-s633 trial. J Clin Oncol 2024;42:410–420. 37963317 10.1200/JCO.23.00975

[26] Min LZ Liu N Zhou Y. 1220P Efficacy and safety of camrelizumab combined with FLOT versus FLOT alone as neoadjuvant therapy in patients with resectable locally advanced gastric and gastroesophageal junction adenocarcinoma who received D2 radical gastrectomy. Ann Oncol 2022;33:S1106.

[27] Bao ZH Hu C Zhang YQ. Safety and efficacy of a programmed cell death 1 inhibitor combined with oxaliplatin plus S-1 in patients with Borrmann large type III and IV gastric cancers. World J Gastrointest Oncol 2024;16:1281–1295. 38660643 10.4251/wjgo.v16.i4.1281 PMC11037035

[28] Cui H Liang W Cui J. Safety and feasibility of minimally invasive gastrectomy after neoadjuvant immunotherapy for locally advanced gastric cancer: a propensity score-matched analysis in China. Gastroenterol Rep (Oxf) 2024;12:goae005. 38425656 10.1093/gastro/goae005 PMC10902683

[29] Lin JL Lin M Lin GT. Oncological outcomes of sequential laparoscopic gastrectomy after treatment with camrelizumab combined with nab-paclitaxel plus S-1 for gastric cancer with serosal invasion. Front Immunol 2024;15:1322152. 38333217 10.3389/fimmu.2024.1322152 PMC10850348

[30] Sun YQ Zhong Q Lv CB. The safety and efficacy of neoadjuvant immunochemotherapy following laparoscopic gastrectomy for gastric cancer: a multicenter Real-world clinical study. Int J Surg 2024;110:4830–4838. 38652275 10.1097/JS9.0000000000001468 PMC11326023

[31] Jiang Q Liu W Zeng X. Safety and efficacy of tislelizumab plus chemotherapy versus chemotherapy alone as neoadjuvant treatment for patients with locally advanced gastric cancer: real-world experience with a consecutive patient cohort. Front Immunol 2023;14:1122121. 37215127 10.3389/fimmu.2023.1122121 PMC10195027

[32] Su J Guo W Chen Z. Safety and short-term outcomes of laparoscopic surgery for advanced gastric cancer after neoadjuvant immunotherapy: a retrospective cohort study. Front Immunol 2022;13:1078196. 36569865 10.3389/fimmu.2022.1078196 PMC9779926

[33] Wang C Wang Z Zhao Y. Neoadjuvant PD-1 inhibitor plus apatinib and chemotherapy versus apatinib plus chemotherapy in treating patients with locally advanced gastric cancer: a prospective, cohort study. J Gastric Cancer 2023;23:328–339. 37129156 10.5230/jgc.2023.23.e17 PMC10154141

[34] Xiong H Li Y. Neoadjuvant PD-1 inhibitor plus apatinib and chemotherapy versus apatinib plus chemotherapy versus chemotherapy alone in patients with locally advanced gastric cancer. Am J Cancer Res 2023;13:3559–3570. 37693166 PMC10492097

[35] Zhang X Zhang C Hou H. Neoadjuvant PD-1 blockade plus chemotherapy versus chemotherapy alone in locally advanced stage II-III gastric cancer: a single-centre retrospective study. Transl Oncol 2023;31:101657. 36934638 10.1016/j.tranon.2023.101657 PMC10034143

[36] Liu Y Li C Lu Y. Tumor microenvironment-mediated immune tolerance in development and treatment of gastric cancer. Front Immunol 2022;13:1016817. 36341377 10.3389/fimmu.2022.1016817 PMC9630479

[37] Naimi A Mohammed RN Raji A. Tumor immunotherapies by immune checkpoint inhibitors (ICIs); the pros and cons. Cell Commun Signal 2022;20:44. 35392976 10.1186/s12964-022-00854-y PMC8991803

[38] Topalian SL Taube JM Pardoll DM. Neoadjuvant checkpoint blockade for cancer immunotherapy. Science 2020;367:eaax0182. 32001626 10.1126/science.aax0182 PMC7789854

[39] Fuchs CS Doi T Jang RW. Safety and efficacy of pembrolizumab monotherapy in patients with previously treated advanced gastric and gastroesophageal junction cancer: phase 2 clinical KEYNOTE-059 trial. JAMA Oncol 2018;4:e180013. 29543932 10.1001/jamaoncol.2018.0013 PMC5885175

[40] Brahmer JR Tykodi SS Chow LQ. Safety and activity of anti-PD-L1 antibody in patients with advanced cancer. N Engl J Med 2012;366:2455–2465. 22658128 10.1056/NEJMoa1200694 PMC3563263

[41] Lin JX Xu YC Lin W. Effectiveness and safety of apatinib plus chemotherapy as neoadjuvant treatment for locally advanced gastric cancer: a nonrandomized controlled trial. JAMA Netw Open 2021;4:e2116240. 34241629 10.1001/jamanetworkopen.2021.16240 PMC8271357

[42] Dos Santos M Lequesne J Leconte A. Perioperative treatment in resectable gastric cancer with spartalizumab in combination with fluorouracil, leucovorin, oxaliplatin and docetaxel (FLOT): a phase II study (GASPAR). BMC Cancer 2022;22:537. 35549674 10.1186/s12885-022-09623-z PMC9097175

[43] Wu L Yan H Qin Y. Fruquintinib plus oxaliplatin combined with S-1 (SOX) as neoadjuvant therapy for locally advanced gastric cancer (GC) or gastro-oesophageal junction adenocarcinoma (GEJ): a multicentre, phase II, single-arm, open-label clinical trial (FRUTINEOGA) protocol. BMJ Open 2024;14:e075696. 10.1136/bmjopen-2023-075696 PMC10862274 38341203

[44] Zitvogel L Galluzzi L Smyth MJ. Mechanism of action of conventional and targeted anticancer therapies: reinstating immunosurveillance. Immunity 2013;39:74–88. 23890065 10.1016/j.immuni.2013.06.014

[45] Ramakrishnan R Huang C Cho HI. Autophagy induced by conventional chemotherapy mediates tumor cell sensitivity to immunotherapy. Cancer Res 2012;72:5483–5493. 22942258 10.1158/0008-5472.CAN-12-2236 PMC4577568

[46] He Q Chen J Zhou K. Effect of additional trastuzumab in neoadjuvant and adjuvant treatment for patients with resectable HER2-Positive gastric cancer. Ann Surg Oncol 2021;28:4413–4422. 33393029 10.1245/s10434-020-09405-6

[47] Li S Yu W Xie F. Neoadjuvant therapy with immune checkpoint blockade, antiangiogenesis, and chemotherapy for locally advanced gastric cancer. Nat Commun 2023;14:8. 36596787 10.1038/s41467-022-35431-x PMC9810618

[48] Jiang H Zheng Y Qian J. Safety and efficacy of sintilimab combined with oxaliplatin/capecitabine as first-line treatment in patients with locally advanced or metastatic gastric/gastroesophageal junction adenocarcinoma in a phase Ib clinical trial. BMC Cancer 2020;20:760. 32795349 10.1186/s12885-020-07251-z PMC7427727

[49] Markham A Keam SJ. Camrelizumab: first global approval. Drugs 2019;79:1355–1361. 31313098 10.1007/s40265-019-01167-0

