# Supplementary material for: The safety and efficacy of neoadjuvant PD-1 inhibitor plus chemotherapy for patients with locally advanced gastric cancer: a systematic review and meta-analysis
Source: Int J Surg. 2024 Aug 22;111(1):1415–26. doi: 10.1097/JS9.0000000000002056 (PMC11745722; doi:10.1097/JS9.0000000000002056)
Supplement: Supplementary file 4 [file js9-111-1415-s004.docx]

**Supplemental material 1** The detailed strategies for literature retrieval.

**Pubmed**

(((((((((((((((((((((((((((((((((((Neoadjuvant Therapy[Title/Abstract]) OR (Neoadjuvant Therapies[Title/Abstract])) OR (Therapy, Neoadjuvant[Title/Abstract])) OR (Neoadjuvant Treatment[Title/Abstract])) OR (Neoadjuvant Treatments[Title/Abstract])) OR (Treatment, Neoadjuvant[Title/Abstract])) OR (Neoadjuvant Chemoradiotherapy[Title/Abstract])) OR (Chemoradiotherapy, Neoadjuvant[Title/Abstract])) OR (Neoadjuvant Chemoradiotherapies[Title/Abstract])) OR (Neoadjuvant Chemoradiation Therapy[Title/Abstract])) OR (Chemoradiation Therapy, Neoadjuvant[Title/Abstract])) OR (Neoadjuvant Chemoradiation Therapies[Title/Abstract])) OR (Therapy, Neoadjuvant Chemoradiation[Title/Abstract])) OR (Neoadjuvant Chemoradiation Treatment[Title/Abstract])) OR (Chemoradiation Treatment, Neoadjuvant[Title/Abstract])) OR (Neoadjuvant Chemoradiation Treatments[Title/Abstract])) OR (Treatment, Neoadjuvant Chemoradiation[Title/Abstract])) OR (Neoadjuvant Chemoradiation[Title/Abstract])) OR (Chemoradiation, Neoadjuvant[Title/Abstract])) OR (Neoadjuvant Chemoradiations[Title/Abstract])) OR (Neoadjuvant Chemotherapy[Title/Abstract])) OR (Chemotherapy, Neoadjuvant[Title/Abstract])) OR (Neoadjuvant Chemotherapies[Title/Abstract])) OR (Neoadjuvant Chemotherapy Treatment[Title/Abstract])) OR (Chemotherapy Treatment, Neoadjuvant[Title/Abstract])) OR (Neoadjuvant Chemotherapy Treatments[Title/Abstract])) OR (Treatment, Neoadjuvant Chemotherapy[Title/Abstract])) OR (Neoadjuvant Systemic Therapy[Title/Abstract])) OR (Neoadjuvant Systemic Therapies[Title/Abstract])) OR (Systemic Therapy, Neoadjuvant[Title/Abstract])) OR (Therapy, Neoadjuvant Systemic[Title/Abstract])) OR (Neoadjuvant Systemic Treatment[Title/Abstract])) OR (Neoadjuvant Systemic Treatments[Title/Abstract])) OR (Systemic Treatment, Neoadjuvant[Title/Abstract])) OR (Treatment, Neoadjuvant Systemic[Title/Abstract])) AND ((((((((((((((((((Neoplasm, Stomach[Title/Abstract]) OR (Stomach Neoplasm[Title/Abstract])) OR (Neoplasms, Stomach[Title/Abstract])) OR (Gastric Neoplasms[Title/Abstract])) OR (Gastric Neoplasm[Title/Abstract])) OR (Neoplasm, Gastric[Title/Abstract])) OR (Neoplasms, Gastric[Title/Abstract])) OR (Cancer of Stomach[Title/Abstract])) OR (Stomach Cancers[Title/Abstract])) OR (Gastric Cancer[Title/Abstract])) OR (Cancer, Gastric[Title/Abstract])) OR (Cancers, Gastric[Title/Abstract])) OR (Gastric Cancers[Title/Abstract])) OR (Stomach Cancer[Title/Abstract])) OR (Cancer, Stomach[Title/Abstract])) OR (Cancers, Stomach[Title/Abstract])) OR (Cancer of the Stomach[Title/Abstract])) OR (Gastric Cancer, Familial Diffuse[Title/Abstract]))

**Embase**

#9. #4 AND #8

#8. #5 OR #6 OR #7

#7. 'neo-adjuvant chemotherapy':ab,ti OR 'neoadjuvant

chemotherapy':ab,ti OR 'neo-adjuvant

therapy':ab,ti OR 'neo-adjuvant treatment':ab,ti

OR 'neoadjuvant treatment':ab,ti OR 'neoadjuvant

therapy':ab,ti

#6. 'neoadjuvant therapy'/exp

#5. 'neoadjuvant chemotherapy'/exp

#4. #1 OR #2 OR #3

#3. 'gastric cardiac cancer':ab,ti OR 'gastric

malignancies':ab,ti OR 'gastric malignancy':ab,ti

OR 'malignancies of the stomach':ab,ti OR

'malignancy of the stomach':ab,ti OR 'malignant

gastric neoplasm':ab,ti OR 'malignant gastric

tumor':ab,ti OR 'malignant neoplasm of the

stomach':ab,ti OR 'malignant neoplasms of the

stomach':ab,ti OR 'malignant tumor of the

stomach':ab,ti OR 'malignant tumors of the

stomach':ab,ti OR 'malignant tumour of the

stomach':ab,ti OR 'stomach cancer':ab,ti OR

'stomach malignancy':ab,ti OR 'stomach

malignancies':ab,ti OR 'pyloric cancer':ab,ti OR

'malignant tumours of the stomach':ab,ti

#2. 'cancer of the cardia':ab,ti OR 'cancer of the

gastric antrum':ab,ti OR 'cancer of the gastric

body':ab,ti OR 'cancer of the gastric

cardia':ab,ti OR 'cancer of the gastric

fundus':ab,ti OR 'cancer, stomach':ab,ti OR

'cardia cancer':ab,ti OR 'gastric antral

cancer':ab,ti OR 'gastric antrum cancer':ab,ti OR

'gastric body cancer':ab,ti OR 'gastric

cancer':ab,ti OR 'gastric cardia cancer':ab,ti

#1. 'stomach cancer'/exp

**Cochrane library**

#1 MeSH descriptor: [Stomach Neoplasms] explode all trees

#2 (Gastric Cancer):ti,ab,kw OR (Familial Diffuse):ti,ab,kw OR (Cancers, Gastric):ti,ab,kw OR (Stomach Cancers):ti,ab,kw OR (Cancer of the Stomach):ti,ab,kw

#3 (Cancers, Stomach):ti,ab,kw OR (Gastric Cancer):ti,ab,kw OR (Cancer, Stomach):ti,ab,kw OR (Cancer, Gastric):ti,ab,kw OR (Cancer of Stomach):ti,ab,kw

#4 (Stomach Cancer):ti,ab,kw OR (Gastric Cancers):ti,ab,kw OR (Neoplasms, Gastric):ti,ab,kw OR (Neoplasms, Stomach):ti,ab,kw OR (Neoplasm, Gastric):ti,ab,kw

#5 (Stomach Neoplasm):ti,ab,kw OR (Neoplasm, Stomach):ti,ab,kw OR (Gastric Neoplasms):ti,ab,kw OR (Gastric Neoplasm):ti,ab,kw

#6 #1 OR #2 OR #3 OR #4 OR #5

#7 MeSH descriptor: [Neoadjuvant Therapy] explode all trees

#8 (Neoadjuvant Chemotherapy):ti,ab,kw OR (Neoadjuvant Chemotherapies):ti,ab,kw OR (Neoadjuvant Chemotherapy Treatments):ti,ab,kw OR (Neoadjuvant Chemotherapy Treatment):ti,ab,kw OR (Treatment, Neoadjuvant Chemotherapy):ti,ab,kw

#9 #7 OR #8

#10 #6 AND #9
