# Supplementary material for: The safety and efficacy of neoadjuvant PD-1 inhibitor plus chemotherapy for patients with locally advanced gastric cancer: a systematic review and meta-analysis
Source: Int J Surg. 2024 Aug 22;111(1):1415–26. doi: 10.1097/JS9.0000000000002056 (PMC11745722; doi:10.1097/JS9.0000000000002056)
Supplement: Supplementary file 5 [file js9-111-1415-s005.docx]

**Supplemental material 2** The outcome indicators utilized in the meta-analyses (NICT/NCT).

| Study (Ref.)  year | pCR | R0 resection | Harvested lymph nodes | Total complications | Severe complications | Postoperative hospital stay (days) | Total TRAEs | Severe TRAEs | 2-year recurrence |
| --- | --- | --- | --- | --- | --- | --- | --- | --- | --- |
| **Phase II/III randomized controlled trials (RCTs)** | | | | | | | | | |
| Shitara ^[22]^  2024 | 52/8 | 321/300 |  |  |  |  | 379/385 | 259/252 | 169/197 |
| Shitara ^[22]^  2024 | 12/4 |  |  |  |  |  |  | 42/21 | 32/45 |
| Lin ^[23]^  2024 | 8/3 | 48/43 | 41.42±19.86/42.83±15.28 |  |  |  |  | 17/14 |  |
| Peng ^[24]^  2024 | 8/3 |  |  |  |  |  |  |  |  |
| Lorenzen ^[25]^  2023 | 35/22 | 135/136 | 59.51±100.33/40.94±52.40 | 64/60 |  |  |  |  |  |
| Min ^[26]^  2022 | 4/1 | 31/21 |  |  |  |  |  |  |  |
| **Retrospective clinical studies (non-RCTs)** | | | | | | | | | |
| Bao ^[27]^  2024 | 4/3 | 23/49 | 27.85±10.27/31.45±12.48 | 5/4 | 1/1 | 11.42±5.67/11.20±4.57 | 21/45 |  |  |
| Cui ^[28]^  2024 | 10/7 | 48/78 | 26.80±10.29/27.59±11.93 | 15/22 | 3/4 | 8.65±2.29/8.35±2.26 | 38/63 | 10/16 |  |
| Lin ^[29]^  2024 | 7/4 | 37/89 | 41.6±18.2/43.3±14.1 | 8/21 | 4/6 | 9.0±5.0/9.2±11.9 |  |  | 13/24 |
| Sun ^[30]^  2024 | 28/25 | 192/383 | 43.97±17.89/39.54±15.01 | 34/63 | 5/9 | 9.11±5.07/9.00±7.43 | 101/177 | 24/45 | 58/159 |
| Jiang ^[31]^  2023 | 13/4 | 50/107 | 28.1±10.3/24.7±9.3 | 13/29 | 1/9 |  |  |  |  |
| Su ^[32]^  2023 | 10/1 | 26/42 | 43.5±14.3/40.0±17.8 | 9/15 | 2/4 | 7.64±2.34/7.00±3.05 | 16/23 | 3/4 |  |
| Wang ^[33]^  2023 | 9/5 | 38/30 |  |  |  |  |  | 8/5 |  |
| Xiong ^[34]^  2023 | 15/8 | 53/44 |  |  |  |  |  | 21/13 |  |
| Zhang ^[35]^  2023 | 8/2 | 33/42 | 25.00±11.43/26.00±12.54 |  |  |  |  | 13/19 | 4/8 |

Abbreviations: NICT, neoadjuvant immunotherapy combined with chemotherapy; NCT, neoadjuvant chemotherapy; pCR, pathological complete response; TRAEs, treatment-related adverse events; RCTs, randomized controlled trials.
