# Supplementary figures and images for: The safety and efficacy of neoadjuvant PD-1 inhibitor plus chemotherapy for patients with locally advanced gastric cancer: a systematic review and meta-analysis
Source: Int J Surg. 2024 Aug 22;111(1):1415–26. doi: 10.1097/JS9.0000000000002056 (PMC11745722; doi:10.1097/JS9.0000000000002056)

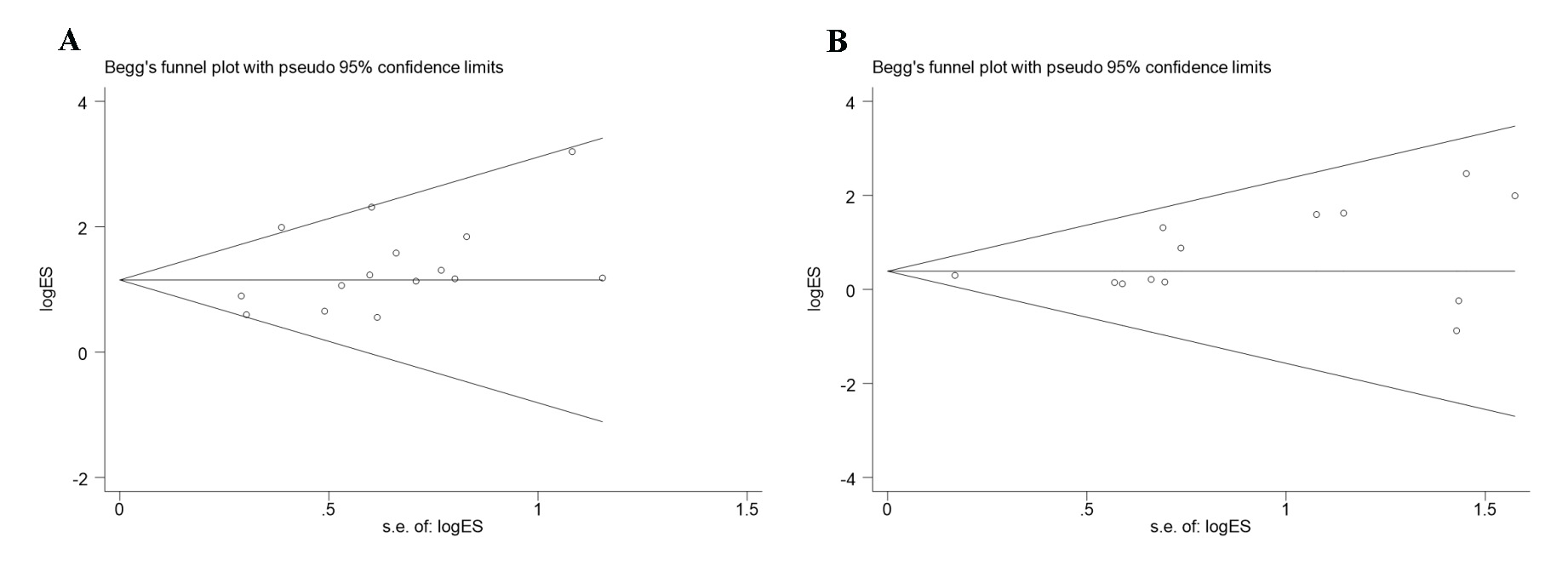

Supplement: Supplementary file 6 [file js9-111-1415-s006.jpg]
